# miR-29b enhances prostate cancer cell invasion independently of MMP-2 expression

**DOI:** 10.1186/s12935-018-0516-0

**Published:** 2018-02-05

**Authors:** Renato F. Ivanovic, Nayara I. Viana, Denis R. Morais, Iran A. Silva, Katia R. Leite, José Pontes-Junior, Gustavo Inoue, William C. Nahas, Miguel Srougi, Sabrina T. Reis

**Affiliations:** 1 Laboratory of Medical Investigation (LIM55), Urology Department, University of Sao Paulo Medical School, Av. Dr. Arnaldo 455, 2° floor, room 2145, Sao Paulo, 01246-903 Brazil; 2 Uro-Oncology Group, Urology Department, University of Sao Paulo Medical School and Institute of Cancer Estate of Sao Paulo (ICESP), Sao Paulo, Brazil

**Keywords:** Prostate cancer, Matrix metalloproteinases, Collagen, microRNA

## Abstract

**Background:**

The ability to metastasize is one of the most important characteristics of neoplastic cells. An imbalance between the action of some matrix metalloproteinases (MMPs) and tissue inhibitors of MMPs drives the invasion process. Some studies have suggested that MMP-2 is involved in metastasis, while other studies have reported that collagen production by cancer cells might also contribute to motility. However, decreased expression of microRNA-29b (miR-29b), which may control MMP-2 and collagen gene expression, has been shown in prostate cancer (PCa). The objectives of the present study were to clarify whether MMP-2 as well as collagens I and III (encoded by COL1A1 and COL3A1, respectively) are controlled by miR-29b and to determine whether metastasis is altered by this relationship.

**Methods:**

PCa DU145 and PC-3 cells were transfected with 100 μL of OPTI-MEM I containing 100 nmol of miR-29b (or its inhibitor) along with 1.5 μL of lipofectamine. Positive and negative controls were prepared using the same protocol. MMP-2, COL1A1 and COL3A1 messenger RNA (mRNA) levels were evaluated via real-time polymerase chain reaction (qRT-PCR). For qRT-PCR, 6 × 10^4^ cells were used. Invasion studies were conducted with Matrigel assays, which simulate invasion of the extracellular matrix by neoplastic cells. After transfection of 3 × 10^4^ cells, invasion was allowed to proceed for 48 h. Invasive cells were counted under an optical microscope. Each experiment was performed in triplicate.

**Results:**

MMP-2 mRNA was not expressed in DU145 cells after transfection with miR-29b. After transfection of cells with the miR-29b inhibitor, COL1A1 (p = 0.02) and COL3A1 (p = 0.06) mRNA expression was increased in DU145 cells, and a large number of transfected DU145 and PC3 cells invaded the Matrigel membrane.

**Conclusions:**

In vitro studies showed that reducing the amount of miR-29b may lead to higher PCa cell invasion via a process that is independent of MMP-2. Collagen expression, controlled by miR-29b, may facilitate this motility process. Thus, the present study suggests that collagen production plays an active role in metastasis control and restoration of miR-29b levels may decrease metastasis. Altogether, these findings support further exploration of drug therapy targeting this aspect of the metastasis circuit.

## Background

Extracellular matrix (ECM) disruption by matrix metalloproteinases (MMPs) is one of the key events in metastasis. MMPs are regulated not only by their natural inhibitors, tissue inhibitors of MMPs (TIMPs), but also at the post-transcriptional level by microRNAs (miRNAs). One of these MMPs is MMP-2, which may be involved in prostate cancer (PCa) progression and metastasis [1, 2].

However, there is evidence that interstitial collagen may be involved in metastasis, indicating an active role for the desmoplastic reaction observed in several cancers. Increased production of several types of collagens has been reported: type II and IV collagens were observed in osteosarcoma [3], collagen type V was produced at elevated levels by fibrosarcoma cells compared with its production in normal muscle cells [4], and increased production of collagens I and III was observed in ovarian carcinoma [5]. Additionally, researchers have reported that collagen expression can facilitate neoplastic cell spreading [6].

The COL1A1 and COL3A1 genes encode the alpha-1 chains of collagen types 1 and 3, respectively, which are present in most connective tissues. Type 1 collagen is present in almost 70% of the extracellular bone matrix. Previously, Steele et al. [7] reported that a single miRNA (miR-29b) regulates MMP-2, COL1A1 and COL3A1 genes, although an assay to evaluate metastasis was not employed. Subsequently, Ru et al. showed that miR-29b overexpression in PCa cell lines limits metastasis, but this study did not focus on collagen genes or MMP-2 and finally Yan et al. [8] employed only LnCaP cells to report that miR-29b upregulation inhibits metastasis and that MMP-2 was not involved in this issue. Therefore, the debate about the relationship between MMP-2, miR-29b, collagen genes and metastases still persists in PCa. Thus, the aim of the present study was to evaluate in vitro whether transfection of PCa cell lines with miR-29b affects metastasis through modification of collagen and MMP-2 gene expression.

## Method

### MicroRNAs

mir-29b, anti-miR-29b and positive and negative controls (Ambion, Austin, TX, USA) were diluted in a 10 μM stock solution and frozen at − 20 °C until further use. All experiments were performed in triplicate.

### Cell lines

The following cell lines were used: DU145 and PC3 (American Type Culture Collection—ATCC). The cells were cultured in DMEM or MEM supplemented with 10% fetal bovine serum (FBS) and 1% antibiotic/antimycotic solution (Sigma Co., St. Louis, MO, USA). Cell cultures were incubated at 37 °C in 95% air and 5% CO_2_.

### Cell transfection

Lipofectamine-based transfection (siPORT NeoFX, Ambion, USA) was performed with 2.5 μL of a 10 μM miRNA stock solution of miR-29b or miR-29b inhibitor. Each inhibitor solution was diluted in 50 μL of OPTI-MEM and mixed with 1.5 μL of Lipofectamine also diluted in 50 mL of OPTI-MEM I. The transfection complex (100 μL) was placed in a 12-well culture plate and incubated for 24 h in CO_2_ at 37 °C. Positive and negative controls were employed in the study. All experiments were performed in triplicate.

### Total RNA and miRNA extraction

At 24 h after transfection, the cells were trypsinized, washed with 10% RPMI and centrifuged at 4000 rpm for 5 min. Total RNA and miRNA were extracted using a mirVana kit according to the manufacturer’s instructions (Applied Biosystems). The purity and concentration of the miRNA and RNA were measured with a spectrophotometer (ND-1000, Thermo Scientific, Wilmington, USA) at wavelengths of 260 and 280 nm (A260/280).

### Reverse transcription (RT)

Reverse transcription was performed using a TaqMan Reverse Transcription kit (Applied Biosystems, Foster City, CA, USA) according to the manufacturer’s instructions. MMP-2 cDNA was synthesized with 5 ng of mRNA (High-Capacity cDNA Reverse Transcription Kit, Applied Biosystems) using reverse transcriptase and random primers.

For miRNA, the reaction was performed with Veriti equipment (Applied Biosystems, Foster City, CA) according to the following parameters: 30 min at 16 °C, 30 min at 42 °C and 5 min at 85 °C. The cDNA from RNA was obtained with same equipment using the following parameters: 10 min at 25 °C, 120 min at 37 °C and 5 min at 85 °C.

### Real-time PCR

We used an ABI 7500 FAST thermocycler system to assess transfection efficacy and the expression of COL1A1/COL3A1 and MMP-2 genes under the following conditions: 0.5 µL of specific primer, 5 µL of TaqMan^®^ Universal PCR Master Mix (Applied Biosystems, California, USA), 3.5 µL of nuclease-free water and 1 μL of cDNA. The PCR cycles were as follows: 2 min at 50 °C, 10 min at 95 °C, and 40 cycles of 15 s at 95 °C and 1 min at 60 °C. The endogenous control was B2M for genes and RNU43 for miRNAs. The miRNA and target gene expression levels were obtained, and the relative quantification of the expression levels was determined using the 2^−ΔΔct^ method [9].

### Invasion assays

Using PC3 and DU145 cell lines, invasion was measured by counting the number of cells that invaded into the lower portion of Transwell chambers (Becton–Dickinson) with a pore size of 8 μm containing 50 mL of Matrigel diluted in serum-free culture medium (1:2). A total of 3 × 10^4^ cells/mL in serum-free culture medium were seeded onto the Matrigel, and 750 μL of culture medium containing FBS (10%) was added to the lower chamber. The cells were maintained in a CO_2_ incubator for 24–32 h at 37 °C. The cells were fixed with formaldehyde in PBS (4%), stained with crystal violet solution (1%) in methanol and counted using an optical microscope at 20× magnification.

## Results

### MMP-2 gene expression

DU145 cells were transfected with miR-29b or its inhibitor. However, there was no MMP-2 amplification. We decided to extract total RNA from DU145 and PC3 cultured cell lines (without any transfection) and from a PCa tissue sample. The MMP-2 mRNA was amplified in the tissue samples but in neither of the cell lines.

### COL1A1 and COL3A1 mRNA expression

**Fig. 1:**
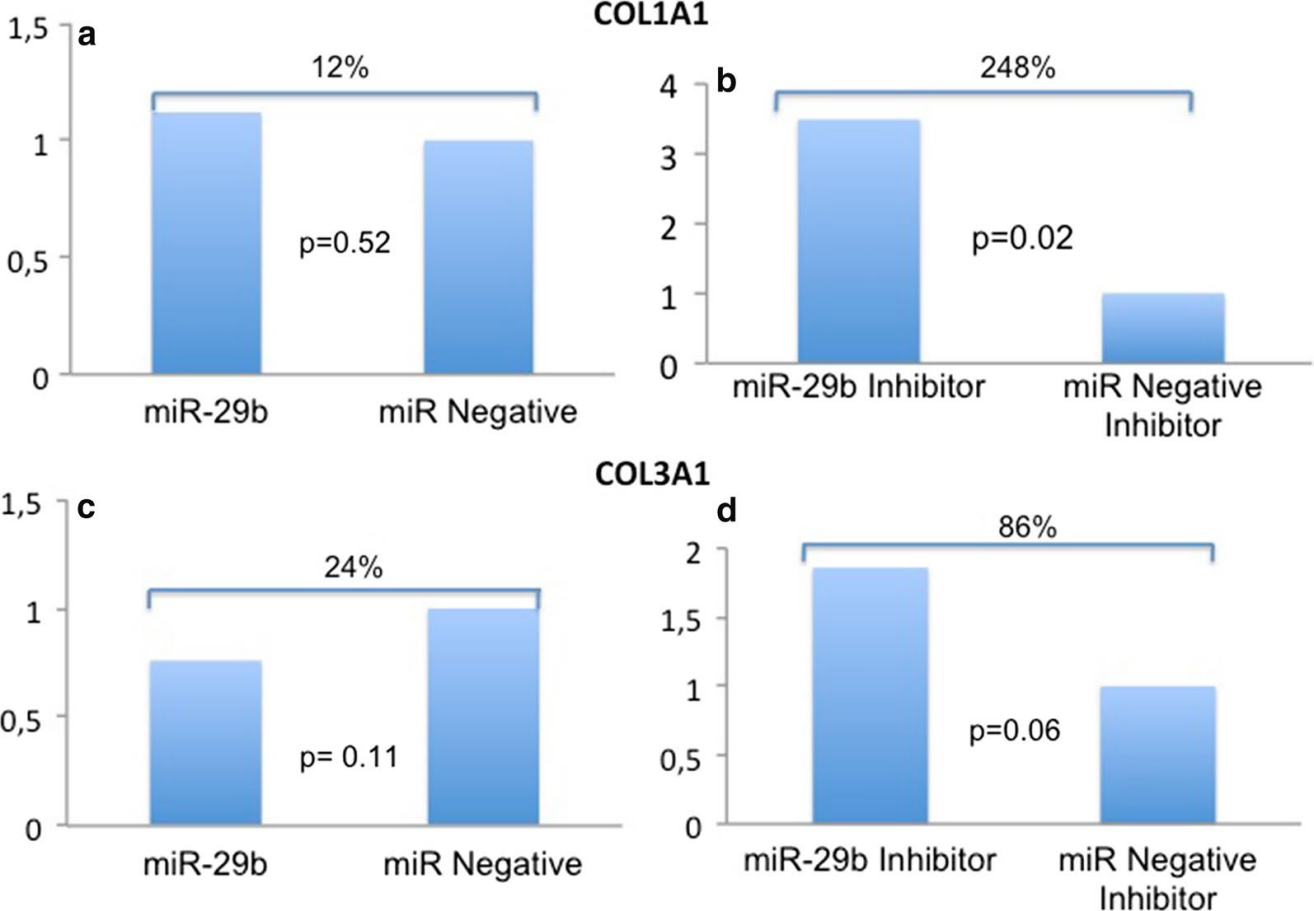
qRT-PCR. There was significant expression of collagen genes after transfection of DU-145 cells with a miR-29b inhibitor. COL1A1 increased 248% (**b**) and COL3A1 86% (**d**) after miR-29b inhibition. However, miR-29b mimics showed no difference in collagen expression (**a** and **c**). The Y axis represent the relative expressions of COL1A1 and COL3A1 mRNAs

In DU145 cell lines, miR-29b inhibition significantly increased COL1A1 (248%, p = 0.02, SD = 1.84) and COL3A1 mRNA levels (86%, p = 0.06, SD = 0.59). However, miR-29 overexpression did not change the expression of those mRNAs (Fig. 1).

### Matrigel assay

**Fig. 2:**
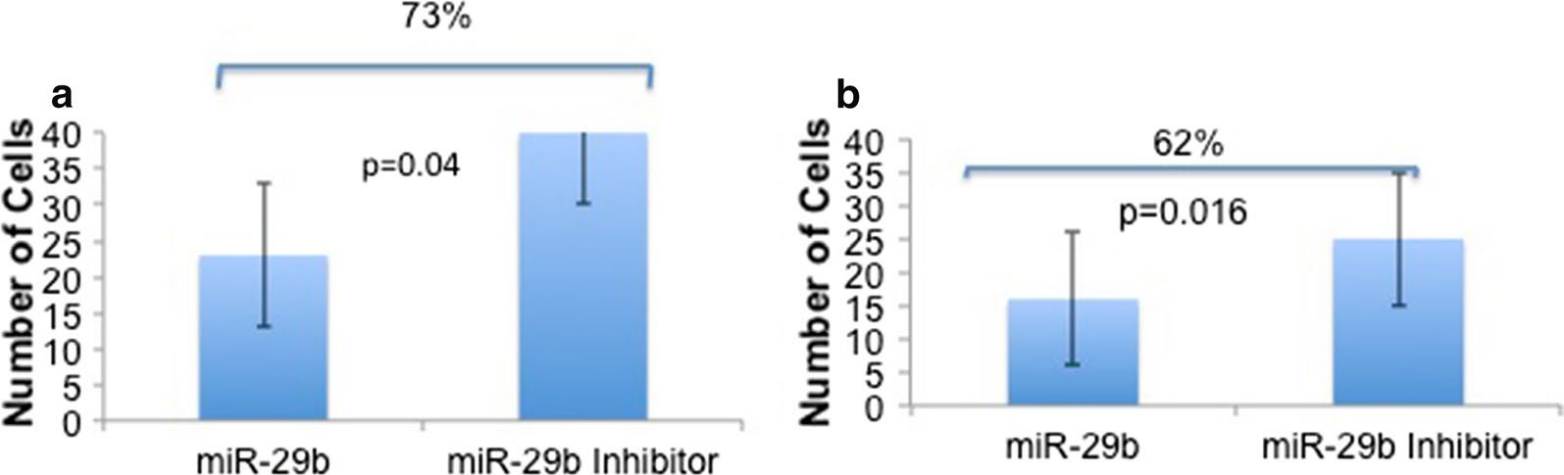
Matrigel experiment. The number of cells that crossed the Matrigel membrane was significantly higher in DU145 (**a**) and PC3 (**b**) cell lines after transfection with the miR-29b inhibitor

**Fig. 3:**
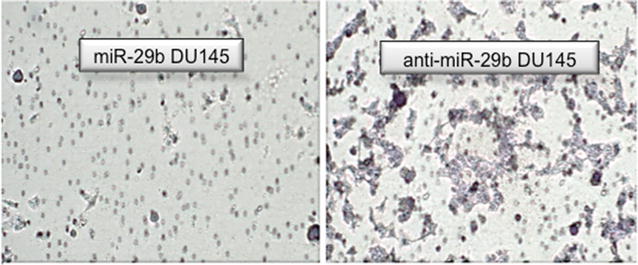
Matrigel chamber photography. Visual representation of the increased invasion of DU145 cells after miR-29b inhibition

miR-29b inhibition increased the number of cells invading through the Matrigel membrane in both the DU145 (p = 0.04) and PC3 cell lines (p = 0.016) (Figs. 2, 3).

## Discussion

Desmoplasia refers to interstitial collagen production in cancer. Collagen types I and III are two of the most important molecules that support tissue structure in solid organs. Decades ago, Waisman et al. [10] observed that the production of extracellular material was an important feature of granulosa-theca tumors, and the culture of chondrosarcoma cells provided evidence of increased collagen production compared to that in normal cells [11].

Richards et al. [12] studied the growth of normal and neoplastic mouse mammary cells on three different substrates: plastic, rat tail, and rat tail collagen. Increased cell growth was reported when collagen was used as a substrate, primarily because the cells produced a structure that resembled collagen IV and laminin. Further, these authors provided evidence that the cells could actively produce collagen, because CIS-OH-proline (which blocks collagen production) inhibited cell growth.

As epithelial cells change their environment, they acquire functions of stromal cells, and collagen production may be one consequence of this transformation [13]. Another example of the interaction between malignant cells and the extracellular environment was provided by Noel et al., who showed higher collagen production in cocultures of MCF7 malignant cells and fibroblasts on a substrate containing type I collagen [14]. However, these authors hypothesized that the collagen was produced not by malignant cells but by fibroblasts. One of the hypotheses regarding the collagen production in neoplastic diseases was that this event functions as an attempt to construct a barrier that can halt tumor spread. However, Hewitt et al. [15] reported that intense collagen production occurred at the center of colon malignancies, in contrast with that observed at the edge where the invasion occurred. These authors, as well as a study published by other authors [14], also considered collagen production by stromal cells. In pancreatic cancer, the desmoplastic reaction occurs with increased production of collagen types I and III, which are primarily stimulated by TGF-β from granulocytes, suggesting an interaction between malignant epithelial cells and the stroma [16]. In renal neoplasms, the myofibroblasts identified in the tumor capsule have high type I collagen mRNA expression, likely also induced by TGF-β [17].

Kaupilla et al. [5] investigated whether fibroblasts or epithelial cells were responsible for the production of collagen types I and III in ovarian cystadenomas and cystadenocarcinomas. These authors demonstrated through in situ hybridization that poorly differentiated tumors could produce type I and III collagens. These authors also highlighted that collagen mRNA does not always translate into fibrils, except in ovarian cancer.

In PCa, there is evidence of collagenous micronodule deposition in biopsy and prostatectomy samples [18]. Further, intense desmoplastic activity was observed in intermediate and high-grade PCa, which also showed increased expression of vimentin, IGF-1, MMP-2, FGF-2, c-Myc, PSCA and Era [19], and an intense reactive stroma has been reported to distinguish benign from malignant prostatic tissue [20]. Such findings highlight the importance of extracellular collagen as a microenvironment component that can enhance metastasis. In other words, changes in the ECM created by tissue fibrosis can enhance tumor progression [21].

Previous studies have shown that malignant cells have reduced levels of miR-29b [22], and other studies have demonstrated reduced miR-29b expression in prostate cell lines and human prostate adenocarcinoma tissues [23, 24].

miR-29b inhibition leads to increased expression of the collagen I, III and V genes in PCa, as reported by Steele et al. [7], and the present results are consistent with these findings. However, in the present study, we found that miR-29b inhibition leads to increased collagen gene expression and further augments metastasis. We would expect that the increased cell invasion in the Matrigel assay was due to MMP-2 overexpression promoted by miR-29b inhibition, because this miRNA was previously shown to control MMP-2. However, because we did not observe MMP-2 mRNA expression in the examined cell lines, it is possible that invasion occurred with collagen gene expression independently of MMP-2. One possible explanation was provided by the results of other studies showing that MMP-2 is primarily expressed by stromal cells, as evidenced by the low to absent MMP-2 levels found in conditioned medium of PCa epithelial cells determined by zymography, whereas stromal cells exhibited higher levels of this protein [25]. Another study conducted by Yan et al. reported decreased cell invasion after miR-29b overexpression only with LncaP cells and without modification of MMP-2 levels. Then, our results obtained with another cell line (DU145) are in line with them [8].

A limitation of the present study is that these results could be improved with ELISA or western blotting to confirm at the protein level the increased collagen production induced by miR-29b downregulation in PCa cells. Another question raised in the present study is whether COL1A1/COL3A1 forms fibrils or if these collagens act as molecules that activate the metastasis pathway. Independently, the observed behavior of PCa cells after miR-29b inhibition indicated increased metastatic potential.

Thus, the present study showed that miR-29b inhibition increases cell invasion, and the expression of COL1A1 and COL3A1 genes in PC3 and DU145 prostate cell lines is independent of MMP-2. Thus, collagen expression may play an active role in metastasis physiology.

## Conclusions

Blockade of miR-29b leads to increased mRNA expression of type I (COL1A1) and type III (COL3A1) collagens and enhances the invasion of PC3 and DU145 cells lines in vitro. This finding provides a possible role for the desmoplastic reaction. This mechanism was independent of the expression of MMP-2, and therapies that restore miR-29b levels might be promising alternatives for controlling the metastatic pathway in PCa.

## Abbreviations

MMP: matrix metalloproteinase
TIMP: tissue inhibitor of matrix metalloproteinase
PCa: prostate cancer
mRNA: messenger RNA
qRT-PCR: quantitative real-time polymerase chain reaction
ECM: extracellular matrix
miRNA: microRNA
miR-29b: microRNA 29b
